# Views and experiences of migrants and stakeholders involved in social and health care for migrants in Italy during the COVID-19 pandemic: a qualitative study

**DOI:** 10.1186/s40359-023-01208-0

**Published:** 2023-05-19

**Authors:** Claudia Lotito, Giulia Turrini, Marianna Purgato, Richard A. Bryant, Mireia Felez-Nobrega, Josep Maria Haro, Vincent Lorant, David McDaid, Roberto Mediavilla, Maria Melchior, Pablo Nicaise, Michela Nosè, A-La Park, Kerry R. McGreevy, Rinske Roos, Andrea Tortelli, James Underhill, Julian Vadell Martinez, Anke Witteveen, Marit Sijbrandij, Corrado Barbui

**Affiliations:** 1 WHO Collaborating Centre for Research and Training in Mental Health and Service Evaluation, Department of Neuroscience, Biomedicine and Movement Sciences, Section of Psychiatry, University of Verona, Verona, Italy; 2 Cochrane Global Mental Health, University of Verona, Verona, Italy; 3 School of Psychology, University of New South Wales, Sydney, Australia; 4 Research, Innovation and Teaching Unit, Parc Sanitari Sant Joan de Déu, Barcelona, Spain; 5 Centro de Investigación Biomédica en Red de Salud Mental (CIBERSAM), Madrid, Spain; 6 Institute of Health and Society (IRSS), Université Catholique de Louvain, Brussels, Belgium; 7 Care Policy and Evaluation Centre, Department of Health, London School of Economics and Political Science, London, UK; 8 Department of Psychiatry, Universidad Autónoma de Madrid (UAM), Madrid, Spain; 9 Centro de Investigaci?n Biom?dica en Red de Salud Mental (CIBERSAM), Madrid, Spain; 10 Hospital La Paz Institute for Health Research (IdiPAZ), Madrid, Spain; 11 Département d’Epidémiologie Sociale, Sorbonne Université, INSERM, Institut Pierre Louis d’Epidémiologie et de Santé Publique, 27 rue de Chaligny, Paris, 75012 France; 12 Department of Clinical, Neuro- and Developmental Psychology, Amsterdam Public Health Institute and WHO Collaborating Center for Research and Dissemination of Psychological Interventions, Vrije Universiteit Amsterdam, Amsterdam, The Netherlands; 13 Research consultant, Brighton, UK

**Keywords:** Migrant, Refugee, Asylum seeker, Qualitative study, COVID-19, Focus group, Free listing interviews, Psychological distress, Mental health

## Abstract

**Background:**

The COVID-19 pandemic has had major and potentially long-lasting effects on mental health and wellbeing across populations worldwide. However, these impacts were not felt equally, leading to an exacerbation of health inequalities, especially affecting vulnerable populations such as migrants, refugees and asylum seekers. Aiming to inform the adaptation and implementation of psychological intervention programmes, the present study investigated priority mental health needs in this population group.

**Methods:**

Participants were adult asylum seekers, refugees and migrants (ARMs) and stakeholders with experience in the field of migration living in Verona, Italy, and fluent in Italian and English. A two-stage process was carried out to examine their needs using qualitative methods including free listing interviews and focus group discussions, according to Module One of the DIME (Design, Implementation, Monitoring, and Evaluation) manual. Data were analyzed using an inductive thematic analyses approach.

**Results:**

A total of 19 participants (12 stakeholders, 7 ARMs) completed the free listing interviews and 20 participants (12 stakeholders and 8 ARMs) attended focus group discussions. Salient problems and functions that emerged during free listing interviews were discussed during the focus group discussions. During the COVID-19 pandemic, ARMs struggled with many everyday living difficulties in their resettlement country due to social and economic issues, revealing a strong influence of contextual factors in determining mental health. Both ARMs and stakeholders highlighted a mismatch between needs, expectations and interventions as factors that may hamper proper implementation of health and social programmes.

**Conclusions:**

The present findings could help in the adaptation and implementation of psychological interventions targeting the needs of asylum seekers, refugees and migrants aiming to find a match between needs, expectations, and the corresponding interventions.

**Trial registration:**

Registration number 2021-UNVRCLE-0106707, February 11 2021.

**Supplementary Information:**

The online version contains supplementary material available at 10.1186/s40359-023-01208-0.

## Background

The COVID-19 pandemic has led to adverse psychological symptoms worldwide [1 − 5]. Prevalence rates of anxiety and depression have increased by almost one third and one fourth, respectively, over the pandemic period [7, 8]. COVID-19 and the associated public health containment measures have had a larger effect on vulnerable populations and people living under fragile socio-economic circumstances, exacerbating already pronounced health inequalities and marginalities in Europe and beyond [9 − 11].

Existing literature suggests that asylum seekers, refugees and migrants (ARMs) are at increased risk of incidence and worsened severity of COVID-19, as well as increased mortality risk [12 − 16]. Compared with the general population, higher levels of psychological distress, with more likely probabilities of depression and/or anxiety, and a lower capacity to access health care services, including mental health care services, have been reported in this population [17].

In order to better identify the needs of vulnerable populations, and to implement actions improving their resilience and mental health, the RESPOND (PREparedness of Health Systems to reduce mental health and Psychosocial concerns resulting from the COVID-19 paNDemic) project has recently been developed [18]. RESPOND is composed of several interrelated work packages focused on the impact of the COVID-19 pandemic on mental health and health inequalities in vulnerable groups, and on the implementation and evaluation of two scalable psychological interventions developed by the World Health Organization (WHO): Doing What Matters in Times of Stress (DWM) [19] and Problem Management Plus (PM+) [20]. DWM is a component of the Self Help Plus (SH+) intervention [21], which is based on acceptance and commitment therapy (ACT) and consists of a pre-recorded audio course delivered by briefly trained facilitators in a group setting and complemented with an illustrated self-help book. DWM is a stress management guide for coping with adversity and aims to equip people with practical skills to help cope with stress. PM + is an individual psychological intervention based on problem-solving and cognitive behavioural therapy techniques for adults impaired by distress in communities exposed to adversity. DWM has been adapted for online delivery as an individually completed self-help intervention with weekly motivational support from a trained helper and combined with PM + into a stepped-care intervention. To be implemented, these two interventions must be adapted to the needs and characteristics of their target populations, incorporating elements that are contextually relevant and meaningful in the culture in which they are being delivered in order to increase acceptability, effectiveness, and participants’ satisfaction [22 − 24].

Qualitative methods may allow to highlight different nuances of important problems, deepening knowledge on different perspectives and views of both end-users and key stakeholders, enabling the collection and integration of the top-down and bottom-up approaches in the design and implementation of mental health programmes. In fact, qualitative studies may help avoid misunderstandings between implementers and partners about what issues programs address, and what approach they take, increasing the amount and depth of information and reflecting the respondent’s own thinking. In addition, qualitative interviewing methods can be used together with quantitative data gathering methods. This study adopted this approach, with a qualitative phase aiming to inform the intervention program design itself. Therefore, the present study was carried out to identify priority mental health views and expectations of migrants, refugees and asylum seekers, as well as key stakeholders with experience in the field of migration, during the COVID-19 pandemic, aiming to have a holistic understanding able to guide the adaptation and implementation of psychological intervention programmes.

## Methods

We conducted a two-stage process qualitative study in the community of Verona (Italy), following the “Module one” of the Applied Mental Health Research Group (AMHR) [25] Development, Implementation, Monitoring, and Evaluation (DIME) manual [26]. During Phase I, we conducted semi-structured Free Listing (FL) interviews where participants were asked to list the problems experienced by migrants, refugees, asylum seekers, and stakeholders since the beginning of the pandemic in Italy. During Phase II, we conducted Focus Group (FG) discussions to gather in-depth information about the problems and functions selected from the FL interview data. Data were analyzed using an inductive thematic analyses approach that primarily use detailed readings of raw data to derive concepts or themes through interpretations made from the raw data by an evaluator, allowing findings to emerge without the restraints imposed by structured methodologies.

### Participants

Eligible participants were adult migrants, refugees, asylum seekers, and stakeholders resettled in Verona, Italy, and fluent in Italian and English (Additional file – Definitions). For stakeholders, we invited mental health professionals with experience both in the field of migration, asylum and integration, working/collaborating with public health services, non-governmental organization (NGO) staff with experience working with migrants, especially with refugees and asylum seekers, and in managing the different phases of the reception system, and cultural mediators with experience in the relationship with migrants.

Project coordinators contacted local non-governmental organizations providing social, health, and/or legal support to migrants, refugees and asylum seekers resettled in Verona to approach potentially interested participants. A snowball sampling approach was subsequently used separately for the two groups to identify additional participants to be involved in the FL interviews, until data saturation (Additional file, Annex I). Gender, background and education level were considered in selecting participants.

During the FL interviews, participants were asked to suggest people knowledgeable about the identified problems, to be invited to contribute to FG discussions. Additionally, if a FL interviewee was particularly knowledgeable about a topic, we invited him/her to attend the FG discussions, where a snowball sampling method was used separately for the two groups until saturation was reached (see Annex I: Sampling Methods). The FG discussions were set up according to an internal homogeneity principle for both separated groups (ARMs and stakeholders); a moderator guided the interview using FL interviews’ lists of problems and functions, and a non-participating observer followed the FG with audio recording tools.

### Data collection

Information was collected in the period between February 2021 and April 2021. Throughout all phases, data were collected by local experienced psychologist interviewers fluent both in Italian and English. The interviewers received training by experienced researchers of the RESPOND project with a background in mental health before each interview phase (FL and FG). This training was integrated with knowledge from scientific web-based sources. In addition, interviewers gained skills on how to accommodate cultural differences in an appropriate manner.

#### Phase 1: Free Listing interviews

The FL interviews consisted of a series of questions asked in a way that generates responses in the form of a list [26]. The purpose of the FL interview was to provide two sets of qualitative data. The first data set represents an overview of all the problems and issues (problems) affecting adult migrants, refugees, asylum seekers, and stakeholders. The second data set is composed of lists of the important daily tasks and activities (functions) that participants regularly do in order to care for themselves and their families, to participate in the community and to deal with problems and issues. Unlike the previous problems data, this information is not used to make planning decisions but to track the impact of interventions. FL interviews were highly structured with single pre-established questions asked to each FL interviewee and a structured data collection form to be completed [26].

Individual FL interviews were conducted in Italian or English via video-calls using an online platform and lasted no longer than one hour. Interviews were conducted by a pair of researchers: one interviewed, while the other one wrote the responses into an interview form (Additional file, Annex III: FL Interview Form and Annex IV: FL Recording Form). Basic pseudonymized information about the respondent was registered, as well as interview details (interviewer, date of the interview and interview ID). An interview ID was assigned by the researcher who kept a secured (digital) document with the identifying key. Interview questions were focused on community views avoiding personal disclosure. A brief questionnaire collected basic demographic data including age, gender, country of origin, and occupation. During the interview, each participant was invited to list all the words that came to mind in response to a series of questions and to provide a brief description for each word. All answers were recorded and transcribed verbatim in the stated order. The questions prompted participants to reflect on topics about both problems and functioning. After completing the FL interviews on problems, the interviewee was asked to respond to primary questions about general functioning.

#### Phase 2: Focus Groups

The FG is a qualitative method for social research, consisting of a discussion between a small group of people, in the presence of one or more moderators and focused on a topic to be investigated in depth [26]. The purpose of these FG discussions was to gather in-depth information about the problems and functions selected from the FL interview data.

FG discussions were implemented using an online platform by two researchers (one who was engaged with the group and one focusing on recording responses) and lasted around two hours each. FGs were recorded on audio, after participants signed the informed consent. Firstly, basic anonymized information about the participant was recorded, as well as discussion details (researcher, FG date and individual ID). An individual ID was assigned by the researcher who kept a secured (password-protected and digital) document with the identifying key. No identifying information was collected at any point during the FGs to ensure anonymity.

FG discussions started with a presentation of the results of the FL problems and functions in order of frequency (most frequent first). The emerging lists of problems and activities were kept separated for ARMs and stakeholders, but presented together in order to identify similarities and discrepancies. FG participants were asked for further description of the problems, actions to manage and to address them (coping strategies and help-seeking behaviour). In addition, they were asked to: identify any important problems (and functions) not present on the list and to add them; prioritise the problems and the activities starting from those considered most important; and identify similarities and discrepancies between ARMs and stakeholder lists in order to clarify different perceptions of problems and solutions (Additional file, Annex V: Focus group form). Then, FG discussions were transcribed for the analysis.

### Data analysis

According to DIME [26], the main objective of the FL data analysis is to consolidate the data into a single list of responses for each FL question. The analysis was conducted on the original data (Italian for stakeholders and English for ARMs) by local interviewers immediately after the interviews, and then translated in English. In order to review and analyse data, the interviewers first consolidated each interview and later they created a master list of responses for each question. Responses were listed and coded without a pre-existing coding framework. When participant responses were similar in meaning but different in wording, they were combined, and the most accurate word or sentence to explain it was chosen.

The result was a list of different problems or functions for each FL question with the ID numbers of interviewees who mentioned each problem. Once all FL interviews were reviewed and consolidated, the interviewers checked the list to ascertain whether any of the responses should be further combined. Finally, the lists were re-organized in order of increasing frequency of the number of interviewees who mentioned each problem or activity by counting up the number of total participants (ID numbers) associated with each item. The frequency of items was used as an indicator of importance.

FG discussions were analysed first by transcribing the audio-recorded discussions, and then by manual open coding of the transcripts to formulate themes. We adopted an inductive thematic analysis [27, 28]. Code words or phrases were applied to sections of text to reliably represent the concepts described by the participants. This was an iterative process performed reading and rereading the participants’ text multiple times. This was conducted independently by two local researchers with a background in psychology. The lists of codes were subsequently shared and ambiguities and discrepancies in coding the qualitative data were discussed and resolved in consultation between both data analysts. Then, similar codewords and phrases were regrouped together and renamed into themes. Results were then organized using the final coded themes, with representative quotations used for illustration. This methodology allowed the participants’ thoughts, words, and experiences to remain central in the pool of findings, and ensured the results to be highly relevant for the aims of this study.

Finally, the Social Ecological Model was used to understand the context of migrants’ wellbeing [29]. The Social Ecological Model assumes that an individual’s well-being and behaviour is influenced by interactions of different levels. The microsystem closest to the individual includes influences, interactions and relationships of the immediate surroundings. The second level is the mesosystem that examines immediate interactions and includes areas such as work, school, church, and neighbourhood. The exosystem does not directly impact the individual, but has effect on the individual, such as community contexts and social networks. The macrosystem includes social, religious and cultural values and influences. Finally, the chronosystem contains internal and external elements of time and historical content.

Participants completed an information sheet and signed an informed consent form before participating in the FL interviews or FG discussion (Additional file, Annex II: Informed Consent Form). The study has been reviewed and approved by the Institutional Review Board (IRB) of the University of Verona (registration number 2021-UNVRCLE-0106707).

### Trustworthiness of study results

A comprehensive understanding of the topics was ensured by: (1) means of data triangulation, as we used two sources of information, ARMs and stakeholders, and (2) by means of methodological triangulation, as two sources of procedures, FL interviews and FG discussions, were employed. Additionally, we performed investigator triangulation: independent researchers completed comparative analyses of individual findings, organized team meetings to compare the analyses, and identified relevant themes.

To enhance the trustworthiness of the study findings, results were shared with study participants at the end of FG discussions, with the opportunity to read and check the data. All participants confirmed that their experience was properly reflected. To minimize personal bias, throughout each phase of the research project the interviewers, who were female psychologists, adopted a reflexive attitude via regular supervision. This was performed to consider how their identities and subjectivities shaped and informed how they were perceived and positioned by the participants. Activities included attendance at project meetings, group reflection, and contemporaneous feedback processes to progress thinking, analysis, and writing, and to generate co-produced knowledge. Ethical considerations included the following: all participants were informed about the study, and it was emphasised that participation was voluntary. Due to ARMs’ unfamiliarity with the research and sensitive nature of the discussions, verbal consent was also obtained from all participants, and reminded them of confidentiality concerns. Refugees and asylum seekers were reassured that refusal to participate or withdrawal from the study would have not affected their legal pathway in any way. Additionally, all study participants were informed that the data collected and study results would not be shared in a way that would allow them to be identified personally in subsequent outputs. Even though the study did not bring an immediate and direct benefit to the participants, we communicated that their engagement was critically important for developing scientific knowledge. Finally, as the research focused on participants’ perceptions and opinions, the risk of harm was deemed to be minimal.

## Results

### Characteristics of the participants

A total of 19 participants (12 stakeholders, 7 ARMs) completed the FL interviews (Table 1).

**Table 1:** Socio-demographics characteristics

	Free listing interviews	Focus groups
	ARMs (N = 7)	ARMs (N = 8)
**Age (years), mean (SD)**	34.57 (7.39)	33.5 (6.50)
**Gender, n (%)**		
Male	5 (71.4)	5 (62.5)
Female	2 (28.6)	3 (37.5)
**Job position**		
No job	1 (14.29)	-
Driver	-	1 (12.5)
Interpreter	1 (14.29)	-
Workman	2 (28.57)	3 (37.5)
Farmworker	1 (14.29)	-
Cultural-mediator	2 (28.57)	3 (37.5)
Assistant chef	-	1 (12.5)
**Country of origin**		
Nigeria	3 (42.86)	4 (50)
Morocco	2 (28.57)	-
Afghanistan	1 (14.28)	1 (12.5)
Pakistan	1 (14.28)	3 (37.5)
**Legal status**		
Documented	7 (100)	8 (100)
	**Stakeholders (N = 12)**	**Stakeholders (N = 12)**
**Age (years), mean (SD)**	32.42 (5.82)	33.3 (6.41)
**Gender, n (%)**		
Male	4 (33.33)	5 (41.67)
Female	8 (66.67)	7 (58.33)
**Job position**		
Mental Health Professionals	3 (25)	4 (33.33)
NGO*s’ legal and social workers	7 (58.33)	5 (41.67)
Linguistic-cultural mediators	2 (16.67)	3 (25)
**Experience in the field of migration**		
Less than 5 years	7 (58.33)	6 (50)
More than 5 years	5 (41.67)	6 (50)

*NGO: Non-governmental organization

Stakeholders were 4 men and 8 women. The age range was 26 to 43. ARMs included 5 men and 2 women with different professional backgrounds. One of them had no job, one was an interpreter, two were workers, one participant was a farmworker and two were cultural-mediators. Their ages ranged from 25 to 46 years. In terms of country of origin, three participants were from Nigeria, two from Morocco, one from Afghanistan and one from Pakistan. The time since resettlement ranged from 2 to 10 years. In terms of their legal status, three participants were regular and documented migrants, three were asylum seekers and one was a refugee.

Twelve stakeholders and eight ARMs attended separated sessions of FG discussion (Table 1). Stakeholders included 5 men and 7 women, with age ranging from 26 to 45 years. ARMs were 5 men and 3 women, with age ranging from 25 to 43 years. In terms of country of origin, four were from Nigeria, one from Afghanistan and three from Pakistan. The time since resettlement ranged from 2 to 10 years. Five participants were regular and documented migrants, one was an asylum seeker and two had a refugee status. In terms of job position, one participant was a driver, three were workers, three were cultural mediators and one was an assistant chef.

Three ARMs and five stakeholders participating in the free listing interviews were considered knowledgeable about the topic and were invited to contribute to FG discussions.

### Free listing interviews

Problems and functions, and stakeholders’ difficulties in working with ARMs.

Salient problems and functions in response to each question are summarised in Table 2.

**Table 2:** ARMs and Stakeholders Perceptions of Problems and Functions

ARMs (n = 7)			Stakeholders (n = 12)					
Question 1: What are all the problems that affect migrants living in Italy since the start of the COVID-19 pandemic?								
**Problems**	**Frequency (N)**			**Problems**			**Frequency (N)**	
Job issue	6			Job issue			11	
Inclusion process	6			Access to services			7	
Financial Issue	5			Digitalization			7	
Distress	5			Document issue			6	
Digitalization	4			Barriers to autonomy			6	
Access to services	4			Freedom restrictions			6	
Freedom restrictions	4			Loneliness and social restrictions			5	
Document issue	2			Understanding public health measures			5	
Housing	2			Sharing physical and emotional spaces			3	
				Relationship with country of origin/community			3	
				Housing			1	
Question 2: Can you think of any other problems that affect migrants living in Italy?								
**Problems**	**Frequency (N)**			**Problems**			**Frequency (N)**	
Document issue	4			Precarious life			9	
Racism	4			Job issue			6	
Emotional distress	4			Racism			6	
Lack of protective environment	3			Document issue			4	
Communication and language knowledge	3			Communication and language issue			4	
Inclusion process and self-determination	2			Relationship with the country of origin			4	
Sharing physical and emotional spaces	2			Housing			4	
				Access to services			3	
				Discrepancy of meaning			3	
				Sharing physical and emotional space			3	
				Transcultural stress			1	
				Hyper-vulnerability			1	
Question 3: What are the activities that migrants frequently do to deal with their problems and to take care of themselves?								
**Activities**	**Frequency (N)**			**Activities**			**Frequency (N)**	
Being engaged in a task	4			Help-seeking from community			8	
Individual resources	4			Individual resources			6	
Help-seeking from community	3			Help seeking from social workers			5	
Be patient and wait	3			Rely on religion and prayer			3	
Respect the rules	2			Live “here and now”			3	
Help-seeking from social workers	2			Alcohol and drug abuse			3	
Cross-cultural mediation	2			Looking for services in the area			2	
Passive attitude	2			Solving concrete problems			1	
Stakeholders (n = 12)								
Question 4: What about the problems that affect people working with migrants since the start of the COVID-19 pandemic?								
Question 5: How do people working with migrants deal with that kind of problem?								
**Problems**		**Frequency (N)**		**Activities**		**Frequency (N)**		
Digitalization		7		Multidisciplinary/team working		4		
Barriers to autonomy process		4		Practical and concrete support		4		
Management of emotional experiences		3		Supporting autonomy process		4		
Managing misunderstanding of public health measures		3		Active listening and acceptance		2		
Intercultural communication		2		Cultural mediation		2		
Institutional disorganization		2		Contextualize expectation		1		
Lack of limits in jobs and hyper-assistance		2		Enhancement of local opportunities		1		
Difficulties in health care treatment		2		Psycho-education		1		
Invisibility and lack of protection		1		Recognize role and limits		1		

* The underlined terms are those mentioned in common by end-users and stakeholders.

Frequency reflects the number of times a term was stated among ARMs and stakeholders in response to a question. The Additional file includes the descriptions of each problem mentioned by participants during the FL interviews and the comparison of salient problems and functions, and stakeholders’ difficulties in working with ARMs. Salient issues and themes that emerged during the FL interviews with ARMs and stakeholders were discussed during the FGs, as reported below.

### Focus group discussions

#### Main themes

During FG, we asked participants to discuss and comment on answers provided not only by stakeholders themselves but also by ARMs, in order to stimulate a discussion on the results and to compare any discrepancies. The main themes emerged during the FG discussion with stakeholders and ARMs are summarised in Table 3 and discussed as follows.

**Table 3:** Focus Groups’ main themes

**Main themes stakeholders**	
*Themes*	*Brief Definition*
**Job Issue**	An immediate and urgent economic issue for migrants, a first step of a long-term project for stakeholders.
**Precarious living circumstances**	Migrants’ living difficulties in the resettlement country, sometimes underestimated by stakeholders.
**Housing and sharing spaces**	Housing as a challenging factor due to administrative and racial issues.
**Isolation during the pandemic**	Social isolation is a major problem for migrants, according to stakeholders.
**Discrepancy of meanings in needs**	The distance between stakeholders and migrants in the recognition of needs, especially in mental health.
**Psychological suffering/distress**	Stakeholders recognized feelings of suffering and pain in migrants during the pandemic.
**Life project concepts**	The post-migratory life project designed for migrants must be shared and sharable to acquire meaning.
**Taking care of oneself**	The discrepancy between the essential activities mentioned by stakeholders and those proposed by migrants.
**Vulnerability**	Perceptions of migrants’ over-vulnerability and “shared vulnerability” as human beings during pandemic.
**Being invisible**	Social workers and all vulnerable groups’ perception of being “invisible” for institutions, during the pandemic.
**Bureaucracy and access to services**	All services limited their access and slowed down their activities, leading to major practical issues for migrants.
**Digitalization**	Online procedures to use and to access services represented a further barrier for migrants.
**Main themes migrants, refugees and asylum seekers**	
*Themes*	*Brief definition*
**Access to services**	The inability to “access services” had immediate consequences on administrative status, preventing them from obtaining regular employment contracts.
**Digitalization**	The “digital revolution” had a positive value because it forces to adapt and to learn new skills.
**Limitation during pandemic**	Movement restrictions and reductions in services reduced opportunities.
**Cultural and linguistic barriers**	The language barrier generates an experience of isolation due to the inability to communicate one’s point of view.
**Racism**	Racism as a manifestation of the difficulty to accept diversity. Being in a vulnerable condition could be circumstances of discrimination.
**Human vulnerability**	On a global level, the pandemic connected Italy with other countries in the world; on a local level it equalizes the local population and migrants as human beings.
**Psychological suffering and distress**	The pandemic generated strong feelings of fear, frustration and confusion, aggravating their already insecure and unstable situation.
**Taking care**	Trying to rely on themselves, on their personal resources and adaptation skills in order to cope with strong feeling and psychosocial problems.
**The role of context**	Obstacles to personal care and to the development of migration projects, on a practical and emotional level.
**Bridging two worlds**	The host country allows practical issues to be resolved (documents, work, housing, education), the context of origin is the ground for emotional and affective support. It is necessary to create a bridge and keep the link between the two worlds alive.

#### Stakeholders

Job Issue. The concept of “job” had different meanings to stakeholders compared to ARMs: if for the latter the job seems to be essentially linked to an immediate and urgent economic issue, for stakeholders it represents the first step of a long-term project. The professionals’ difficulty was to make this project acceptable, because often ARMs could not understand it in the way it was originally conceived for them. In relation to job, a worker said:

“We ask ourselves a series of questions and we see certain problems because we are in a different system of life, because certain primary needs are already satisfied and, therefore, we can take care of other problems and notice other things, which take a backseat when there are no work and no documents” (O6, social worker).

Precarious living circumstances. ARMs often struggle with many living difficulties in the resettlement country - job, housing, documents - leading to inevitable consequences on mental health. The lack of a document, the delay of an appointment, the absence of a response from institutions, could represent a matter of life or death, real or symbolic, with intense emotional reactions. Sometimes stakeholders acknowledge to underestimate ARMs’ essential needs to be satisfied and their attempt to prioritise them, especially while developing projects to improve social inclusion and integration.

Housing and sharing spaces. Basic needs also included finding a home. Housing was a challenging factor due to administrative and racial issues. In some cases, ARMs were forced to live in shared and overcrowded contexts, where living difficulties and conflicts could emerge more frequently.

Isolation during the pandemic. Stakeholders considered social isolation as an important issue for ARMs. Nevertheless, they acknowledged it was not reported as a main problem by ARMs. Regarding this point, a worker said:

“During lockdown I suffered from loneliness, I couldn’t see my family and friends. People with a migration background were required to leave their family of origin and friends. Maybe they have already faced it up. They felt loneliness but maybe in a different way, because in general we always have the possibility to be close to the people we love” (O2, legal worker).

Thus, in stakeholders’ perspective, ARMs had already dealt with the distance from their beloveds, allowing them to tolerate and accept loneliness to an extent during the pandemic.

Discrepancy of meanings in needs. Another emerging theme concerned the distance between stakeholders and ARMs in health needs. Regarding this topic, some professionals acknowledged a difficulty in matching their own and ARMs’ expectations, especially about mental health. Some problems concerned both the different ways to express suffering, and the identification of their actual distress, without anticipating and taking for granted others’ requirements.

“There are categories that are purely ours, I mean Western, and that many times migrants are not able to name as we name them because we invented those names” (O1, social worker). “Maybe we have the inclination…no, surely we have it … to project our categories on migrants, both cultural and personal categories, but the problems migrants perceive are much more concrete, less idealized. Sometimes … More than a priority, mental health needs are our projection because we identify ourselves with the migrant.” (O4, mental health professional).

Psychological suffering/distress. Even though stakeholders found it difficult to combine different perspectives on needs and requirements, they recognized psychological suffering actually exists. They acknowledged pain feelings in ARMs such as sadness, anger, frustration, hopelessness and worries experienced in everyday life.

Life project concepts. The difficulty to combine different perspectives also concerned governmental reception and integration projects for ARMs and the pathways of care within mental health services.

“The reception project is complex and concerns many aspects…health, work, learning the language…it’s a rigid structure that is necessary to live in this context, but it is also necessary from a bureaucratic point of view for reporting. But this rigid structure is not always adaptable to all people…the model we propose is like: learn the language so that you can find a job, so that you can have training and contracts, so then you can have the documents… They are channelled into this track where there are various objectives to be achieved and I think it is a bit suffocating as a structure” (O6, social worker).

In addition, a mental professional stated:

“It is difficult to go further, even if they came for a visit…time after time someone could find a sense in the care we offer, but at the beginning it’s difficult because we are not able to reflect their needs” (O3, mental health professional).

Therefore, stakeholders realised how important it is to signify the project and to make it shareable with ARMs so it can be turned from “theory into practice”. Sometimes, conflicts on expectations could emerge, making it difficult to understand who is lost.

Taking care of oneself. Compared with the way ARMs reported caring for themselves, stakeholders noted that not all the activities matched with those they mentioned. What struck them most was the “waiting for” theme. It does not always coincide with a passive attitude as they thought, but on the contrary it seems to require a great effort to be carried on:

“Waiting and being patient can be seen as a way of caring. We ask them to be patient so many times… I’m surprised that they put it in this section, that they connect it to a kind of exercise of caring and patience” (O1, social worker). This new consciousness had a cathartic value for stakeholders because they realized something they were not completely aware of before: “When I’m in front of someone with a passive attitude I get angry, I get frustrated, but now I realized that this is a way of caring, so I will have a different approach and relate them in a different way” (O5, legal worker).

Vulnerability. Stakeholders also reflected on how often they think about ARMs as a fragile person, thus implementing an assumption of over-vulnerability:

“When I was asked the question, I didn’t think to answer that many people are empowered by telling themselves that they are leaders of their own lives, that they will guide their life, that there is hope and a future.” (O4, linguistic-cultural mediator).

Moreover, the pandemic has created a condition of “shared vulnerability”, involving all human beings. This condition made it possible to establish a greater empathic closeness with the condition of ARMs, opening up to numerous questions:

“If this emergency situation reflected a universal human weakness, how did migrants feel before? Does it mean that vulnerability before was greater on one side than on the other?” (O4 mental health professional).

Being invisible. During the pandemic, social professionals’ perception of their role was “to be invisible” for institutions. This invisibility was manifold: it affected professionals, ARMs and all the vulnerable groups, as the “last of the last”. An example of this perception concerns both the absence of proper prevention and control measures for ARMs, and the lack of protection and guidance for professionals.

Bureaucracy and access to services. Public services do not represent a reference point for ARMs, and all stakeholders agreed on this topic. This was perceived largely during pandemic, when all services limited their access and slowed down their activities, leading to a general disorganisation. These difficulties had consequences on major practical issues for ARMs, such as the renewal and the release of documents, developing strong feelings of frustration, anger and confusion.

Digitalization. Due to the pandemic, “digitalization” of services further complicated access because all activities required online procedures. Not all ARMs had digital literacy and many lacked the possibility to have devices and a good internet connection. For example, distance learning for ARMs families has been a challenge because of the struggle in finding tools and using technology. Digitisation had consequences even in terms of preventing the face-to-face relationships, that are essential in fostering processes of mutual understanding.

#### Migrants, asylum seekers and refugees

Access to services. The first problem ARMs discussed was “access to services” and the immediate consequences on their administrative status (release and renewal of identity documents). Locally, the lack of documents has consequences on the possibility to obtain regular employment contracts and to approach new job opportunities:

“…everything was closed, it was not possible to work…administrative services were almost closed and moreover police headquarters, banks, the post office, the tax agency…everything was closed” (M3, male). Another participant said: “My documents were expiring, I had an appointment at police headquarters but they postponed it, so they expired…without documents I cannot renew my job contract…” (M2, female).

Digitalization. The perception of services’ disorganisation was related to the sudden implementation of digital access. However, “digitalization” also had a positive value. In fact, many recognised the importance of this “digital revolution” because those people who previously had difficulties in using technology were forced to adapt and to learn new skills:

“This is a telematics revolution, thanks to the pandemic. This is a positive aspect that allowed the development of technology in Italy. People who didn’t even know how to turn on a computer or go on the Internet to see their personal files…had to learn how to do it!” (M1, male).

Limitation during pandemic. Another problem concerned movement restrictions within the Italian context and towards the Country of origin. For example, a Moroccan participant said:

“…our COVID-19 deaths were buried here in Italy, without being able to be transferred to the country of origin. Their corpses had to stay here because Morocco didn’t accept them coming from a red zone like Italy” (M6, male). Due to limitations and reductions in services, some migrants were not able to exploit opportunities that had been offered by the Government before the pandemic (e.g., language and profession classes).

Cultural and linguistic barriers. A discussion arose around the language barrier and the experience of isolation that sometimes is generated due to the impossibility to communicate their view point in their native language and adopting their cultural background:

“There are some situations where you feel isolated, in dealing with others to communicate your view point, maybe it’s because the language barrier…also because normally I tend to speak English, even when I speak Italian…I think, I need and I want and always tend to communicate my view point in my own language, in my own ground” (M3, male).

Racism. Racism emerged during individual interviews as a widespread problem, although they reported experiences of racism described as “moderate” in the European context. However, racism is condemned in all its forms mainly as a manifestation of the difficulty to accept diversity:

“…racism is a very heavy word…discrimination hurts a lot, it depends on culture, on life people lead, which can generate this feeling and this behaviour. Unfortunately, it’s a language that we find everywhere, it doesn’t include only one Country, we sometimes find it within the same culture, religion, doctrine. Racism can be named as an element of survival…to survive you have to be very rude, violent, hateful… all these terms can create a vocabulary that is called racism…” (M6, male).

The circumstances in which people live could make the person a victim of racism. Being in a vulnerable situation, like ARMs, could represent one of these circumstances, leading to discrimination experiences:

“not every finger is the same in my hands, similarly all people are also not in the same category. It depends only on circumstances and situations…” (M7, female).

Human vulnerability. The pandemic made the vulnerability “universal and shared” (M6, male). On a global level, it connected Italy with other countries in the world; on a local level it equalizes the local population and ARMs as human beings. In the COVID-19 emergency all people are vulnerable in the same way and unified by the uncertainty of the situation.

Psychological suffering and distress. The pandemic generated strong feelings of fear, frustration and confusion even among ARMs about what was happening:

“both migrants and Italian citizens had not understood the game… we had not understood anything…there is the coronavirus, people are dying, we were scared…” (M5, female).

One participant also reported that coronavirus assumed “the meaning of death”, generating extreme fear. However, during the discussions, it also emerged that the initial confusion about what was happening led some people to distrust the information that media was passing on, producing denialist ideas:

“…but the ways it is here in Italy is making me and other people to be confused … to understand if what is telling us is real or not…” (M4, male). All participants agreed that this emergency situation was an additional source of stress further exacerbating their already insecure and unstable situation: “…there are also your personal problems, your personal issues, your health issues, you are depressed, you are frustrated…” (M7, female).

Taking care. In order to cope with strong feelings and psychosocial problems, the participants stated that they try to rely on themselves, on their personal resources and adaptation skills:

“…because when we decided to leave our country, we were ready to deal with everything that could happen (…) An immigrant who came here is a person who has to sow if he wants to reap. He has to take care of himself, there is no one that gives him anything” (M6, male).

All participants fully agreed on this point:

“it all depends on me, it’s all up to me… you have to settle things by yourself, so you don’t have to depend on anybody” (M1, male) and “It’s a game of your mindset. How do you deal with the situation? How do you face the worst circumstance? It all depends on the human being” (M2, female). Despite the difficult circumstances, they try to “keep them motivated” (M6, male).

The role of context. There were barriers to personal caring and to the development of migration projects. Among these obstacles, participants named both practical aspects, including the lack of documents and the language barrier, and emotional aspects, including feelings of insecurity, fear and uncertainty. Participants emphasised the importance of the context in defining their own well-being.

“The person who has everything and the person who doesn’t have everything… it has an impact on their life and obviously when you don’t get the required answers… It definitely frustrates an individual. For example, if a person doesn’t qualify for their documents, it definitely affects them or the other person… all these things are absolutely relevant because it’s a psychological impact. Certainly, the environment and the situation play a very important role in building the personality of an individual… if you are happy certainly then things will be… I think they will have a new flavour, it will certainly have an impact on your physical life, it will have an impact on your mental life” (M4, male).

When ARMs read the use of drugs and alcohol among stakeholders’ answers, they underlined that this behaviour does not only arise in the host country, but it could already exist in the country of origin:

“It depends on the country and on the environment. As far as I know, immigrants who use drugs and alcohol are not people who use them to forget their problems. Some people were already addicted when they came to Europe and are continuing with the addiction” (M1, male).

During the discussion, some participants agreed that they rely on their social networks for problems that are difficult to manage on their own:

“you have to face small and big problems by yourself, sometimes the situation is out of your control, you need help from the people around, sometimes you need help from the people you are living with” (M2, female).

Bridging two worlds. A migrant who has been resettled for many years with documents and a job introduced the possibility to ask for help from services:

“Fortunately, we immigrants see Italy as a country which can solve our problems, and this is true. We are able to solve our problems in many ways but, if we are not able, there is always someone who gives us help, these people are maybe individuals, or it is the administration, the public services, the institutions” (M6, male).

For the participants, the host country enables them to solve practical aspects (document, job, housing, education). On the other hand, in order to deal with emotional problems, resorting to personal resources also implies a “return to their stranger side” (M3, male), the most familiar for themselves, the most unfamiliar for others in the host country. Their background remains essential whenever they need to obtain support. It is necessary to bridge and keep alive the connection between the two worlds they belong to: the host country and the country of origin:

“…the stranger part stays as a valid part always… for example, prayer is a psychological support to keep me calm. On the other hand, material salvation is given to me by the Italian community…” (M5, female).

Prayer and religion represent important ways to revive their belonging.

Based on the theoretical framework of the Social Ecological Model, the COVID-19 pandemic, as a part of the chronosystem, had a substantial effect among asylum seekers, refugees and migrants [17]. While this group was already confronting with substantial challenges, (i.e. in the access to public services, legal issues, and/or poor housing) the pandemic reshaped many aspects of the daily life. This sociohistorical public health emergency had a crosscutting impact on the well-being of these individuals. The emerged themes occurred at different levels from microsystem to macrosystem. At individual level, the pandemic generated strong feelings of fear, frustration and confusion, aggravating already insecure living situation. At the context level, there have been social, economic, and political measures impacting ARM’s immediate environment. Lockdown measures constituted barriers for communicating with families (also when based in the country of origin), for accessing workplaces and social and legal services, affecting at the same time the mesosystem. The exosystem was also impacted: the reduction of social networks limited the transition process for those planning to reach other countries. Finally, at macrosystem level, ARMs needed to reinforce the bridge between the host country and the country of origin as a way to manage emotional and practical aspects. In addition, the pandemic generated a global and local connection between countries around the world in terms of shared vulnerability as human beings. The interactions and connections between all these levels were recognized to be strong determinants of ARMs’ mental health.

## Discussion

The present qualitative study found that ARMs living in Italy struggled with many living difficulties during the COVID-19 pandemic that were often their main source of concern and distress. These qualitative data are consistent with extensive quantitative data on the stress reported by migrants and refugees during the pandemic [30], with a strong influence of COVID-19 on quality of life and mental health [31, 32].

Frequently stakeholders and institutions neglected the centrality of post-migratory conditions in order to focus exclusively on traumatic events in the pre-migratory process, underestimating the consequences of post-migration living difficulties on mental health. However, based on the existing literature, the post-migration phase in the place of destination and resettlement could increase or worsen social and psychological vulnerability, due to precarious living conditions, social isolation and unemployment, and problems related to applying for asylum [33]. In 2018, the WHO published a technical guidance [34] in order to summarize and propose evidence and policies about mental health in the context of migration. This technical guidance emphasised that high levels of psychological distress in refugees and migrants have been found in numerous studies and systematic reviews [35, 37, 38], even though there is heterogeneity across results [36, 37, 39–41]. Notably, WHO showed that a number of important questions remained unanswered by research, including how and why psychological distress increases after migrants have settled for a long period of time [34, 42]. Refugees who have lived in a host country for more than five years tend to exhibit higher rates of depressive and anxiety disorders than the host population [34, 42]. The literature points out that all vulnerable groups, not just migrants, have a greater risk of worsening their health conditions than those who are less disadvantaged on the social scale [43]. Specifically, migrant populations have a health capital that is destined to be reduced and ultimately to be lost in the long term [44 − 46], due to the accumulation of disadvantages in living and working conditions and to the difficulties in accessing and usability of medical/psychological care [48 − 51] and prevention programs [52]. Thus, the available data suggest that daily stressors exert a direct effect on mental health. By targeting specific stressors deemed as impactful by our participants, we can hypothesize and expect a potential direct benefit of psychosocial interventions in terms of reduced distress and improved psychosocial functioning. More specifically, psychosocial interventions can support mental health by targeting the sources of stress that most immediately affect individuals [33, 53].

The present findings expand and strengthen current knowledge by showing that mental health is influenced by multiple factors, closely linked to each other: socio-economic, cultural and environmental conditions, individual lifestyle attitudes, living and working conditions, as well as social and community influences. In general, the social determinants of health include individual, social and economic aspects that could affect health outcomes [54]. Certainly, these determinants are also responsible for health inequalities within and between countries. As a result, health promotion requires actions to address the full range of potentially modifiable determinants of health [55].

An additional issue that emerged from the present qualitative study concerns the discrepancy in needs - both those anticipated by professionals and those expressed by ARMs and the way they can be met. Often considerable differences in meaning emerged with respect to the main areas of life (e.g., job as an immediate and urgent economic issue vs. a long-term project). We argue that these differences of meaning need to be understood and discussed before implementing an intervention in order to make it acceptable and achievable for ARMs. For instance, the response to psychological suffering that we believe to be appropriate for someone may not always be suitable for someone else, due to different cultural differences. During FG discussions, ARMs often stated how necessary it was to keep alive a connection with their origins in order to deal with emotional issues. Social connections with their homeland represented an anchor they could rely on for support. Meanwhile, they recognised the role of the context in determining their wellbeing and the host country as the place that could potentially respond to their rights.

In this general context, the arrival of COVID-19 pandemic has further worsened previous conditions for the most vulnerable populations, including migrants, refugees and ethnic minorities, both in terms of health and rights, leading to further inequality and marginality [10, 15, 16, 56 − 58]. This study found that stakeholders and ARMs shared an experience of invisibility, feeling as though they were abandoned by institutions in the name of prevention and protection from COVID-19. Even though Italian statistics and health data are difficult to disaggregate by migrant status [59], it is possible that the social and material conditions experienced by different social and racial-ethnic groups lead to a differentiated impacts of the virus in terms of viral spread, symptoms and the evolution of the disease [59]. The interaction between multiple factors - individual pre-existing mental health, as well as ongoing social, political and economical factors - signifies the passage from ‘pandemic’ to ‘syndemic’ [59 − 61], where the theory of syndemics [48] lead us to reflect on how social and political factors could determine, reproduce or exacerbate the clustering of diseases [59].

Overall, our findings revealed important psychosocial problems that affected psychological well-being and mental health of migrants, refugees and asylum seekers. These problems included both basic (physiological, safety) and emotional needs (sense of security, well-being) that need to be considered in the adaptation of DWM and PM + interventions. There is a growing evidence base to support the use of cultural and contextual adaptations to mental health interventions, with the aim to meet unique needs [62]. Adaptation for the trial focused on culturally modified materials or resources, for example adapting vignettes and examples in order to reflect the local life of the target group. Our aim was to improve relatability, incorporating culturally congruent terms/language, or placing emphasis on expectations of the target population. Adaptations were also made to improve the acceptability and suitability of the interventions. This included adaptations of the programmes’ structure by adding specific components in the DWM to increase and facilitate participants familiarity with the intervention approach and concepts, or by varying the length of sessions in the PM+. Therefore, DWM and PM + interventions were revised according to the needs of migrants, refugees and asylum seekers in order to ensure effectiveness, comprehensibility and relevance. Second, since the pandemic has changed the way in which interventions are delivered, especially through the use of digital platforms, DWM was adapted for remote delivery. The use of online tools on the one hand makes it easier to reach people, but on the other hand can highlight a number of access barriers, such as the lack of Internet connection or scarce digital literacy, representing a potential major limitation. Consequently, the developing of this online intervention required work on accessibility through simplifying access, procedures and graphical presentation. Guidance by a helper was also included to support participants in accessing the programme.

Finally, we acknowledge some study limitations. First, including only English-speaking ARMs may have reduced the number of potentially eligible participants, possibly leaving out migrants experiencing higher levels of social vulnerability, language barriers, and leading to rapid data saturation. Second, the small number of participants and the limited variability of their backgrounds restricts the generalizability of the findings to the broader migrant population. However, the aim of the study was not to achieve generalizable results, but to capture the participants’ individual perspectives on a particular topic [63]. Furthermore, the heterogeneity of the profiles involved in both groups, ARMs and stakeholders, may also have influenced the results considering the specificity of problems and needs according to the migratory group and professional profile. Third, ambiguities in linguistic responses were recognised in the analysis and were resolved by discussion between the researchers. However, some nuances of meaning have been lost in the translation process. Finally, due to COVID-19 pandemic restrictions in Italy, FL interviews and FG discussions were conducted online. We acknowledge that this method made it more difficult to interact and involve the participants, as well as to access information related to non-verbal communication, essential to enhance the data collected.

## Conclusions

The COVID-19 pandemic has highlighted the health disparities experienced by socially disadvantaged groups, including ARMs [64]. As a result, it is important to design and implement international, national, and local policies that address these subgroups with populations [65], strengthening the human capital of the individual, increasing social cohesion and proposing a health perspective guaranteed for all but declined to the individual [66].

Our findings have important implications for practice, as they suggest the relevance of developing a critical and reflective approach to enable professionals to question concepts and tools usually adopted in practice and taken for granted, aiming to achieve comparable access and relevance to all people needing mental health assistance [67].

## Electronic supplementary material

Below is the link to the electronic supplementary material.

Additional file: Description of data: additional material related to free list interviews and annexes.

## Data Availability

All data generated or analysed during this study are included in this published article [and its supplementary information files].

## Abbreviations

RESPOND: Improving the Preparedness of Health Systems to Reduce Mental Health and Psychosocial Concerns resulting from the COVID-19 Pandemic
DWM: Doing What Matters in Times of Stress
SH+: Self-Help Plus
PM+: Problem Management Plus
AMHR: Applied Mental Health Research Group
DIME: Development, Implementation, Monitoring, and Evaluation
FL: Free Listing
FG: Focus Group
NGO: Non-Governmental Organisation
IRB: Institutional Review Board
WHO: World Health Organization.
