# Supplementary material for: Views and experiences of migrants and stakeholders involved in social and health care for migrants in Italy during the COVID-19 pandemic: a qualitative study
Source: BMC Psychol. 2023 May 19;11:164. doi: 10.1186/s40359-023-01208-0 (PMC10198022; doi:10.1186/s40359-023-01208-0)
Supplement: Supplementary file 1 — Additional file: Description of data: additional material related to free list interviews and annexes. [file 40359_2023_1208_MOESM1_ESM.doc]

**ADDITIONAL FILE**

[**Definitions** 2](#__RefHeading___Toc103004085)

[**Free listing Interviews: comparison of salient problems and functions, and stakeholders’ difficulties in working with migrants** 7](#__RefHeading___Toc103004086)

[**ANNEX I: SAMPLE METHOD** 9](#__RefHeading___Toc103004088)

[**ANNEX II: INFORMED CONSENT FORM** 10](#__RefHeading___Toc103004089)

[**ANNEX III: FREE LIST INTERVIEW FORM** 16](#__RefHeading___Toc103004090)

[**ANNEX IV: RECORDING FORM** 20](#__RefHeading___Toc103004091)

[**ANNEX V: FOCUS GROUP FORM** 24](#__RefHeading___Toc103004092)

[**ANNEX VI: DESIGN-BASED FOCUS GROUP FORM** 33](#__RefHeading___Toc103004093)

# Definitions

| **Migrant** | The International Organization for Migration (IOM) defines migrant as “*a person who moves away from his or her place of usual residence, whether within a country or across an international border, temporarily or permanently, and for a variety of reasons''* |
| --- | --- |
| **Refugee** | The United Nations High Commissioner for Refugees (UNHCR) defines refugee as “*someone who is unable or unwilling to return to their country of origin owing to a well-founded fear of being persecuted for reasons of race, religion, nationality, membership of a particular social group, or political opinion (…)”* |
| **Asylum seeker** | The United Nations High Commissioner for Refugees (UNHCR) asylum seekers as *“someone whose request for sanctuary has yet to be processed”* |
| **Legend:** International Organization for Migration, Glossary on migration. IML Series No. 34. 2019. <https://publications.iom.int/system/files/pdf/iml_34_glossary.pdf>. Accessed 10 Feb 2022. | |

**Definitions of Free Listing Interviewees terms**

**STAKEHOLDERS_migrants’ problems during pandemic**

| **PROBLEM** | **DESCRIPTION** |
| --- | --- |
| **Job issue** | To lose the job and have difficulties in looking for another occupation. Lack of contractual guarantees. |
| **Limited access to services** | Due to low digital literacy and restrictions in face-to-face activities, ARMs find it difficult to access and use public services, especially health and administrative utilities. |
| **Digitalization** | The digitalization of services represents a further challenge for ARMs with low digital literacy and highlights a gap in possibilities for those who can’t access specific education and tools (devices, internet, etc). |
| **Document issue** | Difficulties in obtaining or renewing documents due to administrative and bureaucratic services interruption or reduced access procedures. |
| **Barriers to autonomy** | Interruption in achieving economic and social autonomy, discontinuity in the getting to know and welcoming process in the host country. |
| **Freedom restrictions** | The impossibility to go out and attend usual social activities prevents ARMs from achieving autonomy and inclusion in the host country. |
| **Loneliness and social restrictions** | Giving up face-to-face social networks leading to heightened sense of loneliness. |
| **Understanding public health measures** | ARMs struggle to respect the rules enacted to prevent the contagion, due to both language and cultural barriers and difficulties in understanding ongoing changes. |
| **Sharing physical and emotional spaces** | Sharing living spaces for a long time involves emotional strains and issues in managing coexistence. |
| **Relationship with country of origin/community** | Impossibility to respect the migratory mandate and to send financial help to the family. Inability to return to the country of origin. Concern for contagions of family members and the possible consequences. |
| **Housing** | Difficulties in finding accomodation. |

**Stakeholders _ problems in general**

| **PROBLEM** | **DESCRIPTION** |
| --- | --- |
| **Precarious life** | Being exposed to precarity and uncertainty (social, economical, juridical, ect) in the host country. Post- migration living difficulties. |
| **Job issues** | Difficulties in finding and accessing job opportunities due to the lack of proper documentation, language barriers, non-recognition of qualification acquired in the country of origin. |
| **Racism** | Perception of being treated differently in comparison to Italians on different levels (institutional, social, etc). Example: different waiting time to book an appointment to gain documents. |
| **Document issue** | Political and institutional barriers to get documents and regular juridical positions. |
| **Communication and language issue** | The lack of knowledge of the Italian language or of some cultural norms of the host country makes the process of reception and inclusion difficult (e.g. the failure to access labor rights, expose subjects to be exploited). |
| **Relationship with the Country of origin** | The relationship with the country of origin becomes complicated whether the ARMs can't respect the mandatory received from the family before moving (e.g. to redeem family conditions, to provide financial support by sending money). |
| **Housing** | Renting a home to ARMs represents a widespread problem due to prejudice and racism. |
| **Access to services** | Access to health and administrative services is made difficult by many barriers: lack of documents, unknowing of language and rights, etc. This failure to access leads to a worsening of living conditions. |
| **Discrepancy of meaning** | In some cases, the discrepancy of life concepts represents an obstacle to the realization of a common project, especially in the reception paths for refugees and asylum seekers. |
| **Sharing physical and emotional space** | The sharing of living spaces, often forced, can cause tensions and emotional reactions, increasing psychological distress. |
| **Transcultural stress** | Traumatic experience due to migration processes can complicate the resettlement, determining situations of distress. |
| **Hyper-vulnerabilitation** | Considering the migrant as hyper-vulnerable and unable to use one's own resources determines the risk of projecting one's expectations onto the other and to avoid building an individualized project shareable and acceptable for the person. |

**ARMs_ problems during pandemic**

| **Job Issue** | Limited job opportunities. Non-renewed contracts due pandemic consequences on specific professional sectors (e.g closure of restaurants and shops). Acceptance of irregular occupation in conditions of exploitation. |
| --- | --- |
| **Inclusion process** | Interruption in achieving economic and social autonomy, discontinuity in the getting to know and welcoming process in the host country. |
| **Financial Issue** | Inability to earn enough money to guarantee a livelihood for oneself and for one's family (in the host country or in the country of origin). |
| **Distress** | The emergency situation generated emotional experiences of anger, concern, confusion, and uncertainty, increasing psychological distress. |
| **Digitalization** | Due to low digital literacy and slight accessibility to devices, digital procedures prevent ARMs from using services properly, worsening social and health conditions. |
| **Access to service** | Delays in releasing and renewing documents related to the pandemic prevented the access to certain services, particularly health services. Due to COVID-19 pandemic, some services were not allowed. Procedures became slower. |
| **Freedom restrictions** | During COVID-19 pandemic, the government has enacted rules to prevent contagion. So, face-to-face interactions were restricted, ARMs were not permitted to go back home and visit their relatives, usual activities were modified. Those changes have been perceived as freedom limitations. |
| **Document issue** | Personal documents and regular juridical position allow to find an occupation and to reach economic independence, as well as to access public services. Consequently, during pandemic any delay or refusal was affecting psychological well being, generating pain and struggle. |
| **Housing** | Difficulties in finding a new accommodation. |

**ARMs _ problems in general**

| **Document issue** | Personal documents and regular juridical position allow to find an occupation and to reach economic independence, as well as to access public services. Consequently, any delay or refusal was affecting psychological well being, generating pain and struggle. |
| --- | --- |
| **Racism** | Experiencing stereotypes and prejudices. Being treated as slackers or parasites. |
| **Emotional distress** | Being tired and overwhelmed by uncertainty for documents. Worried about family in the country of origin. Nervous for precarious living conditions. Nostalgia for their background. Trouble for the traumatic past. |
| **Lack of protective environment** | Despite the sense of gratitude towards the host country, the environment is considered as non-protective. Expectations were not met although all the rules and prescriptions required from the governments have been respected. |
| **Communication and language knowledge** | The knowledge of local language is considered fundamental to establish relationships and avoid misunderstandings. The impossibility to learn it due to political reasons is considered as an obstacle to the inclusion process. |
| **Inclusion process and self-determination** | Interruption in achieving economic and social autonomy, discontinuity in the getting to know and welcoming process in the host country. |
| **Sharing physical and emotional spaces** | Sharing overcrowded places for economic (e.g. saving on rents) or institutional (e.g. characteristics of the reception structure) reasons does not allow to have a human space of privacy and increases the state of tension between housemates. |

# **Free listing Interviews: comparison of salient problems and functions, and stakeholders’ difficulties in working with migrants**

When asked about the problems that have affected migrants living in Italy since the start of the COVID-19 pandemic (**Question 1**), “employment” (Job issue) was the most salient issue shared among the two groups (**Job issue**). During the pandemic, job opportunities were limited, contracts were not renewed and the search for another occupation was extremely difficult. On one hand, stakeholders focused on the lack of contractual guarantees as a barrier to achieve economic and social autonomy, especially for refugees and asylum seekers. On the other hand, ARMs highlighted the need to accept irregular contracts in exploitative conditions, hindering the opportunity to receive government benefits. In addition, they underlined how job issues had both financial and emotional consequences, leading to feelings of uncertainty. Digitalization, limited access to services, and the lack of documentation, were mentioned as overarching and closely related problems by participants. During the COVID-19 pandemic, ARMs found it difficult to obtain or renew documents (**“Document issue”**) due to administrative and bureaucratic interruption of services. Waiting for documents, or in the worst-case scenario having an application rejected, precluded legal recruitment, economic independence as well as access to public utilities, including health services (**“Access to services”**). These terms affected ARMs’ psychological well-being, generating suffering. Low digital literacy represented a further barrier, as online administrative procedures increasingly became the only option to access services. Thus, digitalization (**“Digitalization”**) was recognised as another key obstacle to the inclusion process. ARMs stressed how digitalization represented an unexpected challenge. Due to the government restrictions during the pandemic, some activities (e.g. professional or language classes) have been reduced, slowing down the possibility to learn Italian or to acquire new job skills. Interactions were also limited (**“Freedom restrictions”**), and even documented migrants suffered from being unable to travel to visit their family. According to stakeholders, freedom restrictions forced ARMs to progressively abandon their social network, with negative consequences in terms of increased solitude and loneliness. However, social isolation did not represent a specific problem for ARMs, as stakeholders had supposed it would, leading to a discrepancy between experiences and perceptions. Particularly noteworthy among uncommon problems, difficulty to understand public health measures emerged as a theme that stakeholders experienced with ARMs (“**Understanding public health measures”**). Due to language and cultural problems, ARMs struggled to chase the rapidly changing regulatory measures related to the emergency situation. Thereby, some of them did not respect the rules for reducing the virus contagion, and did not use individual protective devices, as they were not fully aware of the existing measures. Other ARMs experienced the restrictions as a constriction, with confusion, anger, and sometimes denialist ideas. In keeping with the emotional aspect, another problem stated by ARMs concerned how the COVID-19 pandemic impacted on their emotional well-being. Some ARMs reported feelings or fear to be infected and to access the hospital, worries about the pandemic impact on their pre-existing precarious living situation, sense of frustration, concerns for their family in the country of origin, and overwhelming uncertainty feelings (**“Distress”**).

General problems that migrants had to face in Italy (**Question 2**) were similar to those emerged during the COVID-19 pandemic, since the emergency situation only exacerbated them. Three main problems were named in common between the two groups of participants. ARMs were usually exposed to precarious living conditions in the host country. The lack of documents or the delay in obtaining them (**“Document issue”**) represented the main obstacle to their survival and improvement, beyond the pandemic. A further emerging problem was racism (**“Racism”**). On the one hand, stakeholders defined it as being treated differently on institutional and social level in comparison to Italians. An example was the reported different waiting time to book an appointment to gain documents. On the other hand, ARMs reported both the perception to be treated according to stereotypes and considered as profiteers and slackers, who arrived in Italy to do nothing and take advantage of the Government’s benefits. Sharing physical spaces and emotional contexts (**“Sharing physical and emotional spaces”**) was the last common cited problem. Sharing overcrowded places for economic (e.g. saving on rents) or institutional (e.g. characteristics of the reception structure) reasons did not allow having a human space of privacy, increasing the risk of tension between housemates and psychological distress. Therefore, concrete daily life difficulties, including housing, barriers to access services, experiencing different customs and traditions, the changing of social status, along with nostalgia and worries about home in the country of origin, were all constitutive factors of transcultural stress conditions.

When asked what migrants usually do to deal with their problems and to take care of themselves and others (**Question 3**), stakeholders indicated the help seeking from the community as the primary way (**“Help seeking from the community”**). Particularly, ARMs ask for advice and suggestions (e.g. housing) to people already resettled in the country for a while. Moreover, ARMs reported that they used to call relatives, friends or relatives n the country of origin to be supported. Concurrently, they tried to solve their problems relying on their personal resources (**“Individual resources”**) and when they were not able to manage problems by themselves, they asked for concrete support from social and legal workers (**“Help-seeking from social workers”**). According to stakeholders’ experience, ARMs were focused on the present time (**“Live here and now”**- 3), relying on religion and prayers (**“Rely on religion and prayer”**) to solve their problems and receive emotional support. Health professionals considered substance and alcohol abuse (**“Alcohol and drug abuse”**) as a way to escape from everyday suffering. They also interpreted a passive attitude as a defence against a sense of powerlessness. Regarding the same question (**Question 3**), ARMs distinguished between two different activities they usually do to take care of themselves. The first set of activities was related to the COVID-19 pandemic. ARMs talked about using masks, social distancing, and sanitising hands as a way of taking care of themselves. A Moroccan participant reported that the tradition of eating on the same plate or drinking from the same glass could not be followed anymore due to the pandemic. The second set of activities included generic functions that ARMs usually act in their everyday life. Being engaged in a task (**“Being engaged in a task”**) allowed them to deal with problems. Attending professional and educational classes, studying to get a driving licence, learning a new job were all described as helpful activities to keep themselves motivated, avoiding “*negative vibes*” (P1, male). One participant described his own experience emphasising the importance of respecting the rules and (**“Respect the rules”**) relying on personal resources “*to convert theory into practice*”(P3, male): “*Italian Government gives migrants some tools and opportunities to start the knowledge and the inclusion process, but it is also up to the migrant to practice it in order to feel as part of the new context*” (P3, male) (**“Individual resources”**). The community of origin represented a touchstone when ARMs needed support and care. Interactions were maintained also through social networks, which represented an easy way to retrieve lost contacts (**“Help-seeking from community”**). Another important way to care for themselves was defined as “developing patience” (**“Be patient and wait”**). Since many ARMs have been away from their country for a long time and they still live in unstable and precarious conditions, the exercise of patience helped them to tolerate the uncertainty and the frustration of uncontrollable factors. Finally, although ARMs often relied on themselves or on their community in times of need, they recognised the social and legal workers as key figures to address their problems (**“Help-seeking from social-workers”**).

Social/legal workers and health care professionals faced numerous problems in their job, since the start of the COVID-19 pandemic (**Question 4**). The most frequently reported was relative to digitalization of activities (**“Digitalization”**): public services modified their access procedures; vis-à-vis relationships were reduced due to smart-working, making all tasks more complex; ARMs needed to be educated on digital skills. Definitely, forced digitalization due to the pandemic reduced human contacts and slowed down the possibilities of inclusion in the context, in particular for newly arrived migrants. NGOs that manage reception projects for refugees and asylum seekers found it difficult to support people. The loss of in-person relationship reduced “well-established rituals”, such as drinking coffee or eating together, which were considered fundamental socialisation activities (**“Barriers to the autonomy process”**). Another issue was related to the management of misunderstandings of public measures such as the use of masks, social distancing, respecting sanitation and other rules (**“Managing misunderstanding of public health measures”**). This aspect was complicated by the ARMs’ difficulty to understand the evolution of the pandemic, mostly because of language and communication barriers.

Due to forced digitalization, misunderstanding of public measures and administrative issues, stakeholders experienced strong emotions like being under pressure, frustrated, upset and confused. They were compelled to be available “beyond all limits” risking a hyper-assistance attitude. Stakeholders had to manage both their feelings, and the concerns and feelings of ARMs’ (**“Management of emotional aspects”**) (**Question 5**). Multidisciplinary and teamwork may help stakeholders to support each other solving ARMs’ problems. Involving people with different professional backgrounds was required to provide effective and appropriate solutions (“**Multidisciplinary/team working”**). A key figure in the relationship with ARMs are cultural-linguistic mediator (**“Cultural mediation”**) that acts as a “bridge” between people from two different cultures, providing translations and helping to interpret cultural norms and concepts to ARMs. During the encounter with ARMs, stakeholders actively listened (**“Active listening and acceptance”**) to their needs and expectations, offering a progressive concrete aid in order to support their autonomy process (**“Supporting autonomy process”**). In addition, they guided ARMs in the exploration of services and local opportunities, letting them to become more familiar with the context.

# **ANNEX I: SAMPLE METHOD**


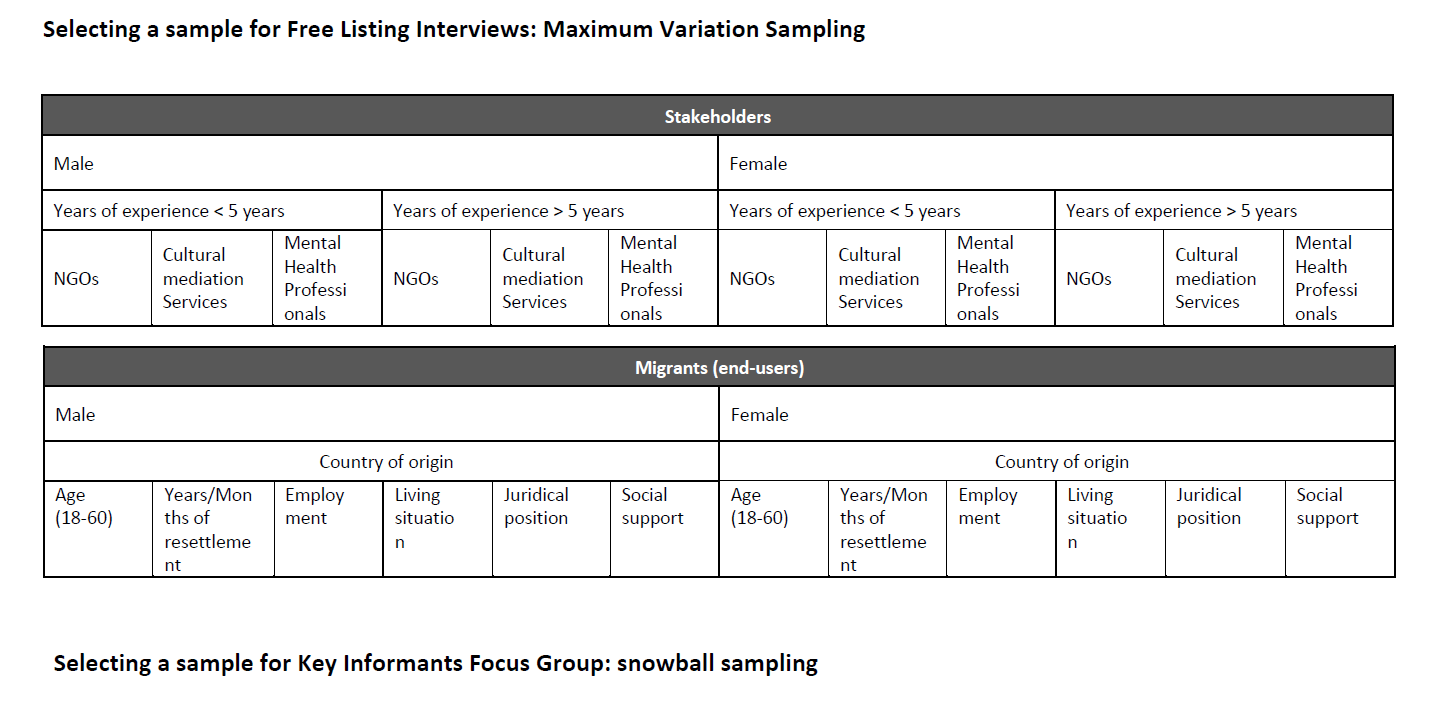


# **ANNEX II: INFORMED CONSENT FORM**

**Informed Consent Form**

**Informed Consent Form for** migrants and stakeholders in Italy who we are inviting to participate in the study titled “**Needs assessment of stakeholders and migrants to integrate the WHO COVID-19 psychosocial support programmes into mental health systems in Italy**”.

**Principal Investigator:** Professor Corrado Barbui, Department of Neuroscience, Biomedicine and Movement Sciences, Section of Psychiatry, University of Verona.

**Organisation:** Department of Neuroscience, Biomedicine and Movement Sciences, Section of Psychiatry, University of Verona.

**Sponsor:** Horizon 2020 (European Commission).

Call: H2020-SC1-PHE-CORONAVIRUS-2020-2.

**Name of the Project:** Improving the PREparedness of Health Systems to Reduce Mental Health and Psychosocial Concerns resulting from the COVID-19 PaNDemic (RESPOND).

**Version:** Version 1.0.

**Date:** December 2020.

**This Informed Consent Form has two parts:**

• **Part 1:** **Information Sheet** (to share information about the study with you).

• **Part 2:** **Certificate of Consent** (for signatures if you choose to participate).

***You will be given a copy of the full Informed Consent Form.**

**Part 1: Information Sheet**

**Introduction**

We work for the Department of Neuroscience, Biomedicine and Movement Sciences, Section of Psychiatry, University of Verona. We are going to give you information about this research study and invite you to participate. You do not have to decide today whether or not you will participate in the research. Before you decide, you can talk to anyone you feel comfortable with about the research. If you have any questions you can contact us at any time and we will be happy to answer them.

**Purpose of the study**

The COVID-19 pandemic has affected countries all over the world, including Italy. The World Health Organisation (WHO) has informed about the potential negative impact of this crisis on mental health and wellbeing of vulnerable populations. We want to find out about the problems affecting migrants and about the tasks and activities they normally do. By learning about these problems and activities, the Department of Neuroscience, Biomedicine and Movement Sciences, Section of Psychiatry, University of Verona, hopes to design better programmes to support migrants during and after the COVID-19 crisis.

**Type of Research Intervention and Participant Selection**

You are being invited to participate in this research as we feel that your experience can contribute to our understanding and knowledge of the problems that frontline health workers are facing because of the COVID-19 pandemic.

**Voluntary Participation**

Your participation in this research is entirely voluntary. It is your choice whether to participate or not and you may change your mind later and stop participating even if you agreed earlier.

**Procedures**

To learn about the problems and activities of migrants during the COVID-19 pandemic, we are interviewing people with a migration background and stakeholders working with this population, who know about the situation. If you agree to be in this study, you will be interviewed by two members of our research team who will ask you some questions about the problems affecting migrants and the things that they do.

During the interviews, we will record basic non-identifying information about you. No identifying information will be collected at any point during the interview to ensure that anonymity is maintained. Next, the interviewers will ask you a series of questions about the problems that migrants are experiencing during the COVID-19 pandemic. You will be asked about the nature of the problems, possible causes, the effects on the person with the problems and others close to them, what people currently do about them and what should be done.

To gather information to design better programmes, the interviewers will ask migrants about the use of technology and how we can use it to design tools to improve the mental health and wellbeing of this people. If you do not wish to answer any of the questions during the interview, you may say so and the interviewers will move on to the next question.

The entire interview will be audio-recorded, but no one will be identified by name on the tape. The information recorded is confidential, and no one else except the members of the research team will have access to it. Once the interview is finished, the recordings will be transcribed and analysed by the research team.

The research is expected to last five months starting from the first interview.

If you are a stakeholder, we will meet you for one interview and one focus group up to two hours each, using online platform (Zoom, Skype, Whatsapp...).

If you are a migrant, we will meet you for one interview and two focus group up to two hours each, using online platform (Zoom, Skype, Whatsapp...).

**Risks**

Talking about problems that affect migrants community can lead to increased stress. However, we will ask you questions about your community or your job, not personal questions about yourself. You will not be asked to share any traumatic events. Furthermore, you may refuse to answer any questions and you may stop the interviews at any time.

**Benefits**

This information will help the Department of Neuroscience, Biomedicine and Movement Sciences, Section of Psychiatry, University of Verona, provide better programmes to improve the mental health and wellbeing of migrants dealing with the COVID-19 pandemic in this area. However, there may be no direct benefit to you personally.

**Compensation**

Your participation in this study will not entail any additional expenditure. If you participate, you will not receive any payment, but you will be offered some small compensation as a thank you for your time. You will receive a voucher worth: 30 €.

**Data Protection and Confidentiality**

Procedures for the collection and storage of personal data will comply with relevant European regulations and directives, in particular with the EU regulation 2016/679 of the European Parliament and of the Council of 27 April 2016 on the protection of natural persons concerning the processing of personal data and the free movement of such data, and the EU General Data Protection Regulation (GDPR), as well as the Organic Act 3/2018 of the 5th of December on personal data protection and the guarantee of digital rights in Italy.

Your data will be processed only to fulfil the purpose for which they were collected and shall be preserved as long as it is necessary following the current regulations. The University of Verona will act as the data controller.

On a general basis, personal data shall not be transferred to third parties, to the extent required by law. You have the right to withdraw your consent at any time. You may exercise the rights of Access, Rectification, Cancellation, Limitation of the treatment, Portability of the data or Opposition to the treatment before the University of Verona (Piazzale L.A. Scuro, 10, 37134 Verona VR), along with a written statement and an identification document.

To safeguard the confidentiality of the participants, data will be safely stored in a locked cupboard at the office of the principal investigator. Data will be coded and the identifying key (a list connecting names to numbers) will be saved on the computer of the research team and secured with a password only known to the principal investigators and the main researchers. Data will only be available to the members of the research group.

**Data sharing**

Only de-identified data will be shared with partners in the RESPOND consortium. The identity of participants in the datasets will be fully safeguarded.

No attributable data will be used in publications. Data will be disseminated through the submission to leading scientific journals with broad dissemination. Also, presentations at international conferences and other external events will be held**.**

**Who to Contact**

If you have any questions, you may contact any of the following: Prof. Corrado Barbui, corrado.barbui@univr.it, Piazzale L.A. Scuro, 10, 37134 Verona VR,

**This proposal has been reviewed and approved.**

**Part 2: Certificate of Consent**

**Title:** “Needs assessment of stakeholders and migrants to integrate the WHO COVID-19 psychosocial support programmes into mental health systems in Italy”.

**Principal Investigator:** Professor Corrado Barbui, Department of Neuroscience, Biomedicine and Movement Sciences, Section of Psychiatry, University of Verona.

**Version:** Version 1.0.

**Date:** December 2020.

**Consent**

**I have been invited to participate in the present study and I hereby declare that:**

· **I have read the information presented in the Information Sheet. I have had the opportunity to ask questions about it and any questions I have been asked have been answered to my satisfaction.**

· **I consent voluntarily to be a participant in this study.**

· **I understand that it is my decision whether or not to be in this study and that I can stop being in this study at any time.**

· **I have read and I agree to the Data Protection Policy presented in the Information Sheet.**

· **I give my permission to audio record my interview for this study and to be accessed solely by members of the research team.**

**Print Name of Participant__________________**

**Signature of Participant ___________________**

**Date ___________________________**

Day/month/year

**Statement by the researcher taking consent**

**I confirm that the participant was allowed to ask questions about the study, and all the questions asked by the participant have been answered correctly and to the best of my ability. I confirm that the individual has not been coerced into giving consent, and the consent has been given freely and voluntarily.**

**A copy of this Information Consent Form has been provided to the participant.**

**Print Name of Researcher taking the consent________________________**

**Signature of Researcher taking the consent__________________________**

**Date ___________________________**

Day/month/year

# **ANNEX III: FREE LIST INTERVIEW FORM**

**Free list interview form**

**Aim:** The Free List (FL) interview consists of a series of questions asked in a way that will generate responses in the form of a list. This method will be used to collect two sets of data: one set of data representing an overview of the problems affecting [target population] (see “Part 1: Problems); and a second set composed of lists reflecting the daily activities that [target population] do to care for themselves and their community (see “Part 2: Function”). This interview is highly structured.

The FL interviews will be conducted by interview pairs: one will interview while the other one records the responses into the interview recording form.

**This document contains the following information:**

***1.*** **Sociodemographic data**

***2.*** **Interview guide**

·

***1.*** **Sociodemographic data**

After introducing yourself, note down in the interview recording form the interview details (interviewer, date of interview, interview ID) and the non-identifying information about the respondent (age, gender, country of origin, role/profession, etc.).

·

***2.*** **Interview Guide**

**Part 1: Problems**

The first set of data represents an overview of all the problems and issues affecting [target population] and stakeholders working with them, in [Italy/France/the Netherlands]. This overview of problems will be written down in the left column of the free listing recording form using the exact language of the interviewee (i.e., no summaries, paraphrasing, or translation).

**Question 1: problem listing**

· **Question 1.1**

***What are all the problems that affect [target population] living in [Italy/France/the Netherlands] since the start of the COVID-19 pandemic?***

The problems included on this list do not all have to be related to mental health, but often there are some issues listed that include mental health and psychosocial concerns.

The interviewee will be repeatedly probed for as many responses as possible by asking the following question:

· **Question 1.2**

**Can you think of any other problems that affect [target population] living in [Italy/France/the Netherlands]?**

· **Question 1.3 (Only for stakeholders)**

**What about the problems that affect people working with [target population] since the start of the COVID-19 pandemic? (Please refer to your job position)**

**The next step is to ask the respondent for a brief description of each problem.**

This description is recorded in the right column of the free listing recording form. Interviewers are instructed to ask for one or two sentences only. If the interviewee provides more than this the interviewer does not attempt to summarize the interviewee’s comments but asks him/her to summarize them.

After the list is complete, the interviewer and recorder review the list of problems and their descriptions, looking for problems potentially relevant to the topic of the study (mental health problems).

**Question 2: identifying key informants**

For each problem listed relevant to the study the interviewee will be asked:

· **Question 2.1**

**Who in the local community do you consider knowledgeable about X?**

(X refers to problems from the list generated by the first question. This question should be asked for every problem identified).

· **Question 2.2**

**Who, if anyone, in the local community would people consult if they had this problem?**

The interviewer records in their notebook the name, role, and contact information of any people who meet these criteria, to contact him/her for attending the focus group.

**Part 2: Function**

The second set of data is composed of lists of the important daily tasks and activities adults regularly do to care for themselves and participate in the community. It is important to make clear that you are asking general questions that refer to end-users and stakeholders in general and not for themselves.

· **Question 1**

***What are the activities that [target population] frequently do to deal with their problems?***

***(Only for stakeholders)* How did people working with [target population] do to deal with that kind of problems?**

· **Question 2**

**What are the activities that [target population] frequently do to care for themselves here in Italy? Would these activities differ by gender?**

· **Question 3**

***What are the activities that [target population] frequently do to care for their community/colleagues here in [Italy/France/the Netherlands]? Would these activities differ by gender?***

· **Question 3**

***What does a [end-user] who is “doing well” look like (describe behaviours/thoughts/mood)?***

· **Question 4**

***What does a [end-user] who is “not doing well” look like (describe behaviours/thoughts/mood)?***

#

# **ANNEX IV: RECORDING FORM**

**Free list recording form: end-users and stakeholders**

**This document contains the following information:**

***3.*** **Sociodemographic data**

***4.*** **Recording form: Problems**

***5.*** **Recording form: Functions**

**3.** **Sociodemographic data**

After introducing yourself, note down the interview details and the following non-identifying information about the respondent.

**Interview details:**

· Interviewer:

· Date of interview:

· Interview ID:

**Sociodemographic information:**

· Age:

· Gender:

· Role/profession:

· Other information:

**4.** **Recording form: Problems**

| **Problems** | **Descriptions** |
| --- | --- |
| 1 |  |
| 2 |  |
| 3 |  |
| 4 |  |
| 5 |  |
| 6 |  |
| 7 |  |
| 8 |  |
|  |  |

**5.** **Recording form: Function**

| **Function** | **Descriptions** |
| --- | --- |
| 1 |  |
| 2 |  |
| 3 |  |
| 4 |  |
| 5 |  |
| 6 |  |
| 7 |  |
| 8 |  |
|  |  |

# **ANNEX V: FOCUS GROUP FORM**

**Key informants focus groups: end-users and stakeholders**

**This document contains the following information:**

*1.* **Sociodemographic data**

*2.* **Interview guide: Part I**

*3.* **Interview guide: Part II**

***1.*** **Sociodemographic data**

After introducing yourself, note down the interview details and the following non-identifying information about the respondent.

**Interview details:**

· Interviewer:

· Date of interview:

· Interview ID:

**Sociodemographic information:**

· Age:

· Gender:

· Role/profession:

· Other information:

***2.*** **Stakeholders’ Focus Group Guide: Part I**

**Aim:** To gather in-depth information about the problems selected from the FL interviews.

**2.1. Introduce the first problem:**

State that other members in the community of [target population] and stakeholders mentioned that the interviewees would be good people to ask about that problem. The interviewer then asks the key informants (KIs) to tell what they know about the problem. Following the introductory question, the interviewer intervenes only to probe for more information on the topic of interest or to guide the interviewee back if they diverge from the topic.

***The interviewer will guide the discussion following these areas:***

**2.2 Nature of the problem:**

Example questions:

· *What are the characteristics/symptoms or signs?*

· *How is X recognized?*

**2.3 Perceived causes:**

Example question:

· *What do [target population] generally perceive as the cause(s) of problem X?*

· *What is/are the cause(s) of problem X?*

***2.4 Effects on the person with the problem and others close to them:***

Example question:

· *What effect does problem X have on the person him/herself?*

· *What effect does problem X have on the people close to that person?*

· *What effect does problem X have on people they work with?*

· *What effect does problem X have in the relationship with stakeholders?*

***2.5 What people currently do about it:***

Example question:

· *Do [target population] seek help for such problems/problem X?*

· If yes: *Where do they seek for help; Do people [target population] receive the help they need?*

· *If no: Why do [target population] not seek for help for such problem(s)?; What are the main reasons [target population] do not seek care?; How could these [target population] best be reached to provide them with health services?*

***2.6 What should be done about it (if they had the resources):***

*Example question:*

· *What should be done about (the effects of) problem X?*

*Repeat the steps for each problem until the respondents has nothing further to say.*

***3.*** **End-users’ Focus Group Guide: Part II**

**Aim:** To gather in-depth information about the interventions to inform adaptation.

**3.1 Introduce this part of the interview:**

*We are also talking to find out ways we can develop tools to help you manage stress.*

*To help us with this, I would like to ask you questions to help us understand more about your situation, your use of technology and how we can use technology to design tools to help manage stress.*

*Please feel free to ask me any questions and only express what you feel comfortable sharing with us. If there are any questions you are uncomfortable with, please let me know. You do not have to answer questions that make you feel uneasy. There are no right or wrong answers, we are interested in your personal opinions and experiences.*

**3.2 Go through the following questions**

| **Tech use section:** Understanding their technology use 10 mins |
| --- |
| 1. *What devices do you have access to?* 2. *What devices have access to the internet? (e.g. WiFI, 3G)* 3. *Do you download any apps?* 4. *What apps do you like, can you give some examples?* 5. *How and when do you use video sites like YouTube?* 6. *How and when do you use social media? (e.g. Facebook, Twitter, Instagram)* 7. *Can you remember the last time you used technology to learn something? When was it? Where were you? What were you learning? What device?* |

| **Help-seeking section:** to get information on the use of online help and support |
| --- |
| 1. *Have you ever accessed information on mental health or stress management? How did you access that information (e.g. online, visited a professional, talked to a friend, etc.)?* 2. *Have you ever used a self-help or online course? When was it? Where were you? What were you learning? Can you say a little about what you liked or did not like about it?* 3. *Are there any online materials (videos, apps, text, etc.) that you like and that helps you manage stress?* 4. *Have you ever taken part in an online health consultation? Can you say a little about what you liked or did not like about it?* 5. *What techniques have you tried to support your emotional wellbeing / reduce stress before (include here activities such as relaxation, as well as social support and other approaches)?*    - *Which ones have been helpful and why?*    - *Which ones have been unhelpful and why?*    - *Have you heard or tried?*   ü Meditation.  ü Talking with member of you community.  ü Planning nice activities (e.g. seeing friends) when you do not feel like it.  ü Praying.  ü Listening to music.  ü Doing physical exercise and activities.  ü Being kind to yourself (self-compassion).  ü Identifying and acknowledging your values and what you do to live by them.  ü Others.  *f.* *Do you use anything that you have found useful regularly? What encourages you to use techniques that you find helpful to manage stress?* |

| **Suggestions for online support system:** To gather ideas on adaptations for the stepped care model |
| --- |
| *We are looking to develop an online self-help intervention for stress that will be deliverd through computers or smartphones. This will be in the form of an app or an online course. I would like to ask you some ideas on how to present this. After people have completed the self-help intervention if they are still experiencing problems with the stress, they can be referred for an online consultation.*  *Questions on access*  Have you heard of any similar interventions for being used by people with a migration background? (if so which?)  Are there any barriers you can think of that may make it difficult for people to access this intervention?  (Interviewer: probe for access difficulties due to internet, access to smartphones, reluctance to use such an intervention etc)  *I would like to show you some example content for your input and review (*e.g. present DWM illustrated guide and introduce this to the user). Ask the following questions as you go through the content:  a) Broadly speaking, is there anything you have seen in the review which you or your community may find:  · Unacceptable or offensive?  · Not relevant to your situation or culture?  · Difficult to understand?    b) Can you say a bit more?  c) What would you propose we do to address these problems?  d) Can you tell me what you like about this book/content?  e) Can you tell me what you do not like about this book/content?  · What would you propose we change? |

# **ANNEX VI: DESIGN-BASED FOCUS GROUP FORM**

**Design-based key informants Focus Groups form**

**Aim:** To gather in-depth information about the interventions to inform adaptation

**This document contains the following information:**

***1.*** **Sociodemographic data**

***2.*** **Interview guide**

***1.*** **Sociodemographic data**

After introducing yourself, note down the interview details and the following non-identifying information about the respondent.

**Interview details:**

· Interviewer:

· Date of interview:

· Interview ID:

**Sociodemographic information:**

· Age:

· Gender:

· Role/profession:

· Other information:

***2.*** **Interview guide:**

**Example introduction:**

*Our team are talking to people with migration background to find out ways we can develop tools to help you manage stress. To help us with this, I would like to ask you questions to help the team understand more about your situation, your use of technology and how we can use technology to devise tools to help manage stress.*

*Please feel free to ask me any questions and only express what you feel comfortable sharing with us. If there are any questions you are uncomfortable with, please let me know. You do not have to answer questions that make you feel uneasy.*

*There are no right or wrong answers – we are not here to test your knowledge in any way, we are interested in your personal opinions and experiences.*

**Go through the following questions:**

| ***1.*** **Tech use section:** Understanding their technology use 10 mins |
| --- |
| 1. *What devices do you have access to?* 2. *What devices have access to the internet? (WiFI, 3G, where)* 3. *Are there any restrictions to your internet access? For example certain times of day, limited data etc. (interviewer – ensure you collect detail on any restrictions including times where access is possible and specifics of limitations. In particular it is important to document if the user does not have access to the internet at certain hours or for periods of time).* 4. *Do you download any apps? (if no, explore why apps are not downloaded)* 5. *What apps do you like, can you give some examples?* 6. *How and when do you use video sites like YouTube?* 7. *How and when do you use social media? (facebook, twitter)* 8. *Have you ever used video conferencing or calls 9e.g. Zoom, Skype etc).*    1. *How often and for what purposes?*    2. *Was it video or calls?*    3. *When was the last time you used this and for what purpose (e.g. social, educational, medical etc)?* 9. *Can you remember the last time you used technology to learn something? – when was it? Where were you? What were you learning? What device?* |

| ***2.*** **Help seeking section:** to get information on use of online help and support |
| --- |
| 1. *Have you ever accessed information on mental health or stress management? How did you access that information (e.g. online, visited a professional, talked to a friend etc).* 2. *Have you ever used a self-help or online course? when was it? Where were you? What were you learning? Can you say a little about what you liked or did not like about it.* 3. *Are there any online materials (videos, apps, text etc) that you like and that helps you manage stress?* 4. *Have you ever taken part in an online health consultation? Can you say a little about what you liked or did not like about it.*      1. *What techniques have you tried to support your emotional wellbeing / reduce stress before (include here both activities such as relaxation as well as social support and other approaches)?*    - *Which ones have been helpful and why?*    - *Which ones have been unhelpful and why?*    - Have you heard or tried:   ü Meditation.  ü Talking with member of you community.  ü Planning nice activities (e.g. seeing friends) when you do not feel like it.  ü Praying.  ü Listening to music.  ü Doing physical exercise and activities.  ü Being kind to yourself (self-compassion).  ü Identifying and acknowledging your values and what you do to live by them.  ü Others. |

| **Suggestions for online support system:** To gather ideas on adaptations for the stepped care model |
| --- |
| *We are looking to develop an online self-help intervention for stress that will be deliverd through computers or smartphones. This will be in the form of an app or an online course. I would like to ask you some ideas on how to present this. After people have completed the self-help intervention if they are still experiencing problems with the stress, they can be referred for an online consultation.*  *Questions on access*  Have you heard of any similar interventions for being used by people with a migration background? (if so which?)  Are there any barriers you can think of that may make it difficult for people to access this intervention?  (Interviewer: probe for access difficulties due to internet, access to smartphones, reluctance to use such an intervention etc)  *I would like to show you some example content for your input and review (*e.g. present DWM illustrated guide and introduce this to the user). Ask the following questions as you go through the content:  f) Broadly speaking, is there anything you have seen in the review which you or your community may find:  · Unacceptable or offensive?  · Not relevant to your situation or culture?  · Difficult to understand?    g) Can you say a bit more?  h) What would you propose we do to address these problems?  i) Can you tell me what you like about this book/content?  j) Can you tell me what you do not like about this book/content?  What would you propose we change? |

| **Prioritisation exercise** |
| --- |
| *If we support this content with a trained helper contacting you weekly (for up to 15 minutes), what activities would you like to do in that call/meeting? Please order in terms of priority:*  a) Practice exercise (e.g. breathing to reduce stress).  b) Answer any questions about the techniques.  c) Help to apply exercises to my situation.  d) General listening to my problems.  e) Other (specify). |
